# Deficiency of Rbpj Leads to Defective Stress-Induced Hematopoietic Stem Cell Functions and Hif Mediated Activation of Non-canonical Notch Signaling Pathways

**DOI:** 10.3389/fcell.2020.622190

**Published:** 2021-01-25

**Authors:** Ram Lakhan, Chozha V. Rathinam

**Affiliations:** ^1^Institute of Human Virology, University of Maryland School of Medicine, Baltimore, MD, United States; ^2^Center for Stem Cell and Regenerative Medicine, University of Maryland School of Medicine, Baltimore, MD, United States

**Keywords:** hematopoiesis, hematopoietic stem cells, notch signaling, Hes1, stress hematopoiesis

## Abstract

Deregulated notch signaling has been associated with human pathobiology. However, functions of notch pathways in hematopoiesis remain incompletely understood. Here, we ablated canonical notch pathways, through genetic deletion of Rbpj, in hematopoietic stem cells (HSCs). Our data identified that loss of canonical notch results in normal adult HSC pool, at steady state conditions. However, HSC maintenance and functions in response to radiation-, chemotherapy-, and cytokine- induced stress were compromised in the absence of canonical notch. Rbpj deficient HSCs exhibit decreased proliferation rates and elevated expression of p57^Kip2^. Surprisingly, loss of Rbpj resulted in upregulation of key notch target genes and augmented binding of Hes1 to *p57* and *Gata2* promoters. Further molecular analyses identified an increase in notch activity, elevated expression and nuclear translocation of Hif proteins, and augmented binding of Hif1α to *Hes1* promoter in the absence of Rbpj. These studies, for the first time, identify a previously unknown role for non-canonical notch signaling and establish a functional link between Hif and Notch pathways in hematopoiesis.

## Introduction

Notch signaling pathway is highly conserved and plays a vital role in development and adulthood. There are essentially four classes of molecules; Notch receptors, ligands, positive, and negative regulators, and transcription factors, that control notch signaling network (Pajcini et al., 2011; Lobry et al., 2014). Mammalian system contains four Notch receptors (Notch1-4) that can be activated by five ligands (Delt-like 1, 3, and 4 and Jagged 1 and 2) (Gordon et al., 2008; Kopan and Ilagan, 2009; Radtke et al., 2010). Upon engagement with its ligand, from the neighboring cell, the extracellular domain of notch receptor induces a conformation change that ultimately results in liberation of the intracellular domain (ICD) of Notch receptor (NICD) and subsequent translocation to the nucleus. Under the canonical scheme of notch signaling, NICD heterodimerizes with the DNA binding transcription factor-recombination signal binding protein for immunoglobulin k J region (Rbpj, also known as CSL/CBF-1) and recruits additional co-factors, including mastermind proteins (MAML 1–3), to activate target genes, such as members of the Hairy enhancer of split (Hes) and Hairy related (Hey or Hrt) gene families (Gordon et al., 2008; Kopan and Ilagan, 2009; Radtke et al., 2010; Pajcini et al., 2011; Lobry et al., 2014).

Loss of functions studies have indicated the possible involvement of Notch signals in hematopoiesis (Radtke et al., 2010; Pajcini et al., 2011; Bigas and Espinosa, 2012). In particular, notch signals are essential for intra-embryonic hematopoiesis (Robert-Moreno et al., 2005), emergence of definitive hematopoietic stem cells (HSCs) from endothelial cells (Kumano et al., 2003), and maintenance of fetal liver HSC pool and functions (Gerhardt et al., 2014). Within the adult hematopoietic system, notch signals control T cell development in the thymus (Pui et al., 1999; Wilson et al., 2001; Chen et al., 2019), differentiation of M1 Vs. M2 macrophages (Wang et al., 2010), Megakaryocyte development (Mercher et al., 2008), Erythrocyte differentiation (Oh et al., 2013) and Dendritic cells (Caton et al., 2007; Kirkling et al., 2018). However, functions of notch in the maintenance of adult HSCs remain controversial.

Earlier studies, including our own (Rathinam et al., 2006), established that augmented notch signals through retroviral mediated overexpression of notch in both human and mouse HSCs causes immortalization and/or unlimited expansion without compromised self-renewal under *in vitro* culture conditions (Pui et al., 1999; Varnum-Finney et al., 2000; Stier et al., 2002; Duncan et al., 2005; Francis et al., 2017). In agreement with these data, we have shown that physiological events leading to accumulation of notch1 levels in HSCs, due to loss of a E3 ubiquitin ligase-itch, results in enhanced HSC maintenance and functions (Rathinam et al., 2011). These findings have unequivocally demonstrated the gain-of-functions role of notch signals in HSCs. Surprisingly, a series of loss-of-functions studies, through retroviral mediated overexpression of the dominant negative (dn) form of MAML (Maillard et al., 2008; Benveniste et al., 2014), Vav^cre^ mediated genetic activation of dnMAML (Francis et al., 2017) and MX1^cre^ mediated deletion of Jagged1, *Notch1*, *Notch2*, and *Rbpj* (Mancini et al., 2005; Maillard et al., 2008; Varnum-Finney et al., 2011), suggested a dispensable role for notch in the maintenance of adult HSCs. Taken together, these studies established that exaggerated notch signals play key roles in HSCs, even though its deficiency may not affect HSC physiology.

To understand the complex roles by notch pathway in HSCs and explain this molecular paradox, we ablated Rbpj mediated canonical notch signals in HSCs and studied the downstream consequences on HSC maintenance and functions. Our data specify that canonical notch signals play indispensable roles in the differentiation of lymphoid-primed multipotent progenitors (MPP4) and hematopoietic recovery following radiation-, genotoxic- and cytokine- induced stress. Unexpectedly, our studies identified that Rbpj deficiency leads to activation of notch target genes through Hif1α mediated non-canonical notch pathways in HSCs.

## Materials and Methods

### Mice

Rbpj Floxed mice (Han et al., 2002) (kind gift of Dr. Tasuku Honjo), Vav-iCre (B6.Cg-Commd10^Tg(Vav1–icre)A2Kio^/J) mice and R26-CreERT2 (B6.129-*Gt (ROSA) 26Sor^*tm*1*(cre/ERT2)Tyj*^*/J) mice were purchased from the Jackson Laboratory. CD45.1 congenic animals were purchased from the National Cancer Institute. The Institutional Animal Care and Use Committee approved all mouse experiments.

### Cell Preparation

Mice were analyzed between 4–12 weeks after birth, unless otherwise specified. RBCs were lysed with ammonium chloride (STEMCELL Technologies). Trypan blue (Amresco)–negative cells were counted as live cells.

### Flow Cytometry

Cells were analyzed by flow cytometry with Attune Nxt (Thermofisher) and FlowJo software (Tree Star). The following monoclonal antibodies were used: anti- CD34 (RAM34), anti-CD45.1 (A20), anti-CD45.2 (104), anti-CD48 (HM48-1), anti-CD117 (2B8), anti-Flt3 (A2F10.1), anti-Sca-1 (D7), anti-B220 (RA3-6B2), anti- CD19 (1D3), anti-CD3 (145-2C11), anti-CD4 (GK1.5), anti-CD8 (53-6.7), anti-CD11b (M1/70), anti– Gr-1 (RB6-8C5), and anti-Ter119 (TER119; from BD Biosciences); anti-CD150 (TC15-12F12.2) from Biolegend; anti-CD16/32 (93) from eBioscience. Cells incubated with biotinylated monoclonal antibodies were incubated with fluorochrome-conjugated streptavidin–peridinin chlorophyll protein–cyanine 5.5 (551419; BD), streptavidin-allophycocyanin-Cy7 (554063; BD), streptavidin-super bright 650 (Biolegend). In all the FACS plots, indicated are the percentages (%) of the gated fraction.

### BMT Experiments

1 × 10^6^ of bone marrow cells were injected into lethally irradiated (10 Gy) congenic (CD45.1^+^) recipient mice. For competitive-repopulation experiments, 5 × 10^5^ BM cells from either control or KO mice were mixed with 5 × 10^5^ of WT (CD45.1^+^) BM cells (to obtain a ratio of 1:1) and were injected into lethally irradiated congenic WT (CD45.1^+^) recipient mice.

For serial transplantation assays, 1 × 10^6^ of bone marrow cells were injected into lethally irradiated (10 Gy) WT congenic (CD45.1^+^) recipient mice. After 12 weeks of transplantation, 1 × 10^6^ BM cells of primary recipients were injected into lethally irradiated WT congenic secondary recipients.

### RNA Extraction, PCR, and Real-Time PCR

Total RNA was isolated with an RNeasy Mini kit or RNeasy Micro kit (QIAGEN). cDNA was synthesized with Oligo (dT) primer and Superscript IVReverse Transcriptase (Thermo Fisher Scientific). PCR was performed with T100 thermal cycler (Bio-Rad Laboratories) and TSG Taq (Lamda Biotech). Real-time PCR was performed in duplicates with a CFX-connect real-time PCR system (Bio-Rad Laboratories) and SsoAd- vanced SYBR Green Supermix (Bio-Rad Laboratories) according to the manufacturer’s instructions. Relative expression was normalized to the expression levels of the internal control (housekeeping gene) HPRT/GAPDH.

### ChIP Assay

ChIP assay was performed with Pierce Agarose ChIP Kit (Pierce) according to the manufacturer’s instructions. In brief, 5 × 10^6^ Lineage negative BM cells were fixed and immunoprecipitated with anti-Hes1 (abcam ab49170) and anti-Hif1 α (Novusbio NB100-105) antibody or control-IgG antibodies. Immunoprecipitated DNA fragments were quantified by real-time PCR with the use of primers which amplify Gata2 and p57 promoter regions containing Hes1 binding sites and Hes1 promoter region containing Hif1 α binding sites. Fold enrichment was normalized to goat IgG-precipitated samples.

### Western Blot Analysis

For immunoblot analyses, cells were lysed with cell lysis buffer (Cell Signaling Technology) with protease inhibitor cocktail (Complete; Roche) and 1 mM PMSF (Santa Cruz Biotechnology, Inc.). Cell lysates were boiled with sample buffer (NuPAGE; Life Technologies) containing 1% 2-Mercaptoethanol (Sigma-Aldrich). In some experiments, cytoplasmic and nuclear proteins were fractionated using Subcellular Protein Fractionation Kit (Thermo Fischer). Proteins were subjected to 8–12% SDS-PAGE and transferred to PVDF membranes (Bio-Rad Laboratories). The membranes were blocked with either 5% bovine serum albumin (Life Technologies) or 5% skim milk and then treated with primary and secondary antibodies, respectively. The blots were visualized using the ECL (Pierce) and C300 (Azure Biosystems) western blot imaging unit. Antibodies used were as follows: anti-P57 (Novusbio NBP2-44488), anti-Hes1 (abcam ab49170), anti–ICN1 (Novusbio NB100-78486), anti-actin (I-19; Santa Cruz Biotechnology, Inc.), anti-Histone3 (Cell Signaling Technology 96C10) HRP-conjugated anti–mouse and anti–rabbit IgG (Cell Signaling Technology), and HRP-conjugated anti–goat IgG (Santa Cruz Biotechnology, Inc.).

### Measuring ROS Levels

BM cells were stained with cell surface markers and then incubated with 2 mM CM-H_2_DCFDA (Life Technologies C6827) in pre-warmed HBSS at 37C for 15 min. The cells were then pelleted and resuspended in PBS before acquisition.

### *In vitro* Culture

Lineage depleted BM cells were placed in culture with 10%FBS/DMEM + 50 ng/ml stem cell cytokines (Flt3l, SCF, TPO, IL3, and IL6; Peprotech). After indicated period of culture, cells were harvested, stained with antibodies and analyzed by flow cytometry.

### Confocal Microscopy

Freshly sorted Lin- BM cells were plated onto poly-D-lysine (Sigma) coated chamber slides and incubated at 37°C in 10%FBS/DMEM + 50 ng/ml stem cell cytokines (IL3, SCF, TPO, Flt3l, and IL6) for 2 h-overnight before fixing for 10 min with 4%PFA at RT. Cells were permeabilized in 0.15% Triton-X100 for 2 min at RT and then blocked overnight in 1%BSA/PBS at 4°C. Cells were incubated with primary in blocking solution for 2 h at 37°C and then with secondary for 1 h at 37°C. For nuclear stain, Sytox blue (Thermofisher) was added for 6 min at RT and then slides were mounted with VectaShield and imaged on a Zeiss 710 Confocal using a 100× objective. Florescence quantification was performed by ImageJ (NIH) analysis similar to Mccloy et al., 2014 except that the values for the background were obtained from the surrounding cells and *p*-values were obtained by performing a two-tailed unpaired Student’s *t*-test. For these experiments, cells stained only with secondary were used to determine the specificity and intensity of fluorescence.

### 5-FU and Cell Proliferation Assay

A single dose (150 mg/kg) of 5-Fluorouracil (5-FU; Sigma-Aldrich) was injected intraperitoneally and mice were analyzed after 14 days. For *in vivo* bromodeoxyuridine (BrdU) assay, 1 mg BrdU (BD) was injected intraperitoneally. After 24 h of injection, mice were sacrificed and bone marrow cells were stained for BrdU, following the BrdU Flow Kit manufacturer’s instructions (BD Pharmingen). For Ki67 analysis, BM cells were stained for cell surface markers, fixed, and permeabilized with BD Fix/Perm kit. Cells were stained with anti-Ki67—APC (BD558615) for 30 min on ice and analyzed by flow cytometry.

### Statistics

Data represent mean and SEM. Two-tailed student’s *t*-tests were used to assess statistical significance (^∗^*P* < 0.05, ^∗∗^*P* < 0.01, ^∗∗∗^*P* < 0.001). For survival curve analysis, log rank test was used to assess statistical significance (^∗^*P* < 0.05, ^∗∗^*P* < 0.01, ^∗∗∗^*P* < 0.001, ^****^P < 0.0001).

## Results

### Loss of Rbpj Results in Altered MPP Pool

**FIGURE 1:**
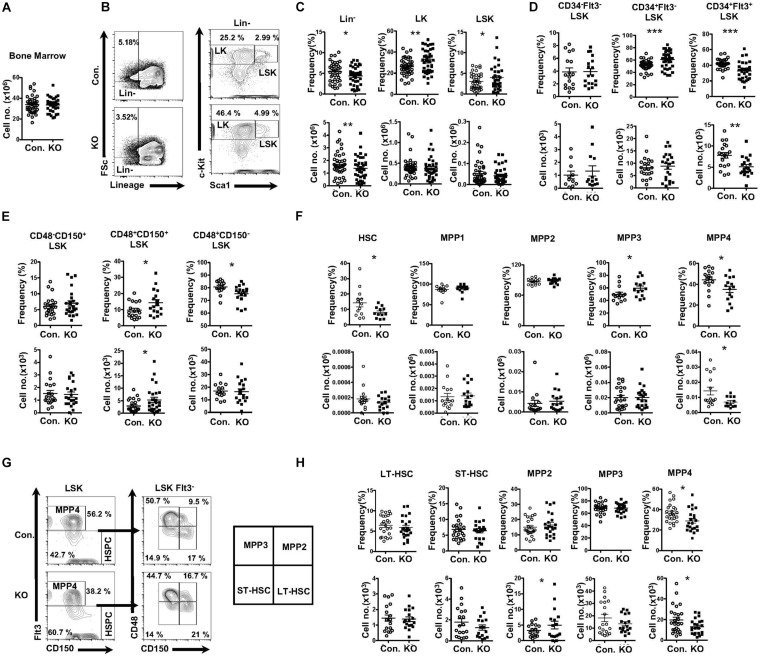
Deficiency of Rbpj affects lymphoid- and erythroid- biased progenitors. **(A)** Absolute cell number of BM from KO and Control mice (*n* = 35–40). **(B)** FACS plots of Lin^–^, LK, and LSK subsets of BM (two femurs and two tibias) from KO and control mice. Data are representative of seven independent experiments. **(C)** Frequencies (top) and absolute numbers (bottom) of Lin-, LK, and LSK subsets of BM (two femurs and two tibias) from KO and control mice (*n* = 16–25). **(D)** Frequencies (top) and absolute numbers (bottom) of LT-HSC, ST-HSC, and MPP subsets of BM (two femurs and two tibias) from KO and control mice (*n* = 16–25). **(E)** Frequencies (top) and absolute numbers (bottom) of CD150^+^CD48^–^LSK (LT-HSC), CD150^+^CD48^+^LSK and CD150^–^CD48^+^LSK subsets of BM (two femurs and two tibias) from KO and control mice (*n* = 16–25). **(F)** Frequencies (top) and absolute numbers (bottom) of HSC, MPP1, MPP2, MPP3, and MPP4 analysis of BM (two femurs and two tibias) from KO and control mice (*n* = 14–20). **(G)** FACS plots of LT-HSCs, ST-HSCs, MPP2, MPP3, and MPP4 subsets of BM (two femurs and two tibias) from KO and control mice. Data are representative of seven independent experiments. **(H)** Frequencies (top) and absolute numbers (bottom) of LT-HSCs, ST-HSCs, MPP2, MPP3, and MPP4 subsets of BM (two femurs and two tibias) from KO and control mice (*n* = 16–25). All data represent mean ± SEM. Two-tailed Student’s *t-*tests were used to assess statistical significance (*P < 0.05; **P < 0.01; ***P < 0.001).

To study the role of Rbpj mediated signals in the hematopoiesis, we analyzed Rbpj^F/F^ Vav^cre/+^ mice (henceforth referred to as Rbpj^Hem–KO^), as expression of cre through Vav promoter results in faithful deletion of transgenes in all hematopoietic cells, including LT-HSCs. To investigate the contribution of Rbpj mediated canonical notch pathway to the maintenance of HSPC pool, we enumerated the frequencies of HSPC subsets in the bone marrow (BM) of Rbpj^Hem–KO^ mice. Our analysis of Rbpj^Hem–KO^ mice indicated normal cellularity of the BM (Figure 1A). Immunophenotyping studies on HSPCs of BM documented a decrease in the relative frequency, but normal absolute numbers, of lineage^–^ (Lin^–^) hematopoietic cells, and a relative increase, but normal absolute numbers, of Lin^–^ c-Kit^+^ Sca1^+^ (LSK) cells of Rbpj^Hem–KO^ mice (Figures 1B,C), as reported earlier (Duarte et al., 2018). To assess if Rbpj deficiency affects the distribution of specific HSPC subsets, we further analyzed the BM LSK cells of Rbpj^Hem–KO^ mice through previously established immunophenotyping strategies; (1) according to the scheme established Yang et al. (2005) we identified normal relative and absolute numbers of LT-HSCs (CD34^–^Flt3^–^LSK), increased relative, but normal absolute numbers of Short-Term HSCs (ST-HSCs; CD34^+^Flt3^–^LSK), and decreased relative and absolute numbers of Multipotent Progenitors (MPPs; CD34^+^Flt3^+^LSK) (Figure 1D and Supplementary Figure 1A); (2) immunophenotyping approach of Oguro et al. (2013) revealed normal relative and absolute numbers of LT-HSCs (CD150^+^CD48^–^LSK), increased relative, but normal absolute, numbers of CD150^+^CD48^+^LSK subset, and decreased relative, but normal absolute, numbers of CD150^–^CD48^+^LSK subset (Figure 1E and Supplementary Figure 1B); (3) HSPC characterization, established by Wilson et al. (2008) identified normal relative and absolute numbers of HSCs (CD34^–^Flt3^–^CD150^+^CD48^–^LSK), modestly increased relative, but normal absolute, numbers of MPP1 (CD34^+^Flt3^–^CD150^+^CD48^–^LSK), normal relative and absolute numbers of MPP2 (CD34^+^Flt3^–^CD150^+^CD48^+^LSK), modestly increased relative, but normal absolute, numbers of MPP3 (CD34^+^Flt3^–^CD150^–^CD48^–^LSK), and a consistent decrease in both relative and absolute numbers of MPP4 (CD34^+^Flt3^+^CD150^–^CD48^–^LSK) (Figure 1F and Supplementary Figure 1C); and (4) through an immunophenotyping scheme established by Pietras et al. (2011) we identified that the relative and absolute numbers of LT-HSCs (Flt3^–^CD150^+^CD48^–^LSK), ST-HSCs (Flt3^–^CD150^+^CD48^–^LSK), and granulocyte/monocyte lineage biased MPP3 (Flt3^–^CD150^–^CD48^+^LSK) fraction were normal. Normal relative, but increased absolute, numbers of megakaryocyte/erythroid lineage biased MPP2 (Flt3^–^CD150^+^CD48^–^LSK) fraction were identified. Consistent with the other HSPC identification strategies, both relative and absolute numbers of the lymphoid biased MPP4 (Flt3^+^CD150^–^CD48^+^LSK) fraction were decreased (Figures 1G,H). Taken together, these data unequivocally demonstrate that Rbpj deficiency selectively affects the differentiation and/or maintenance of the MPP subsets, under steady state conditions.

### Rbpj Deficiency Leads to Defective Erythroid and Lymphoid Differentiation

**FIGURE 2:**
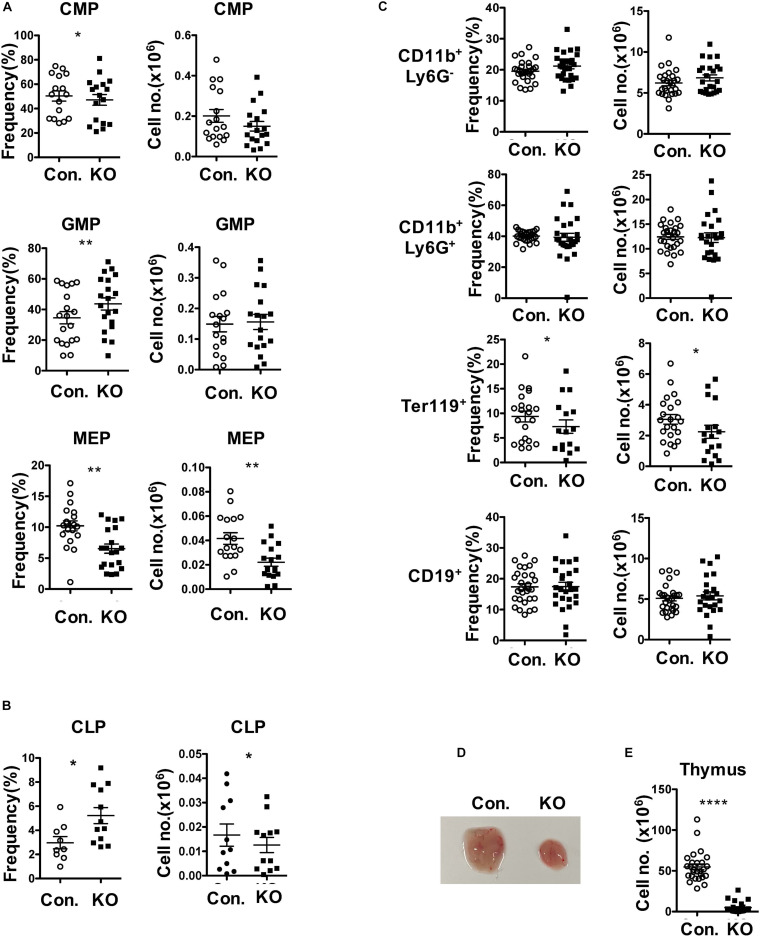
Loss of Rbpj affects lymphoid and erythroid differentiation. **(A,B)** Frequencies (left) and absolute numbers (right) of CMPs, GMPs and MEPs **(A)** and CLPs **(B)** of BM (two femurs and two tibias) from KO and control mice (*n* = 10–20). **(C)** Frequencies (left) and absolute numbers (right) of CD11b^+^Ly6G^–^, CD11b^+^Ly6G^+^, Ter119^+^, and CD19^+^ subsets in the BM of (two femurs and two tibias) from KO and control mice (*n* = 12–25). **(D,E)** Representative picture showing size **(D)** and cellularity **(E)** of thymus from KO and control mice (*n* = 25–30). All data represent mean ± SEM. Two-tailed Student’s *t-*tests were used to assess statistical significance (*P < 0.05; **P < 0.01; ****P < 0.0001).

To assess the functions of Rbpj mediated notch signals and impact of altered MPP pool in Rbpj^Hem–KO^ to multi-lineage differentiation, we determined the frequencies of myeloid-, erythroid- and lymphoid- lineage cells in Rbpj^Hem–KO^ mice. Analysis of lineage committed progenitors (Figures 2A,B and Supplementary Figures 2A,B), revealed an increase in relative, but normal absolute, numbers of Common myeloid progenitors (CMPs; Lin^–^Sca1^–^c-Kit^+^ CD34^+^CD16/32^–^) and Granulocyte/Monocyte Progenitors (GMPs; Lin^–^Sca1^–^c-Kit^+^ CD34^+^CD16/32^+^). However, both relative and absolute numbers of Megakaryocyte/Erythroid Progenitors (MEPs; Lin^–^Sca1^–^c-Kit^+^ CD34^–^CD16/32^–^) were decreased. Analysis of Common Lymphoid Progenitors (CLPs; Lin^–^IL7Rα^+^Sca1^low^c-Kit^low^) revealed that their relative frequencies were increased, but absolute numbers were decreased. Next, enumeration of myeloid, erythroid and lymphoid lineage cells in BM of Rbpj^Hem–KO^ mice indicated (Figure 2C) normal frequencies and absolute numbers of CD11b^+^ myeloid- and CD19^+^ B- lineage cells, and reduced frequencies and absolute numbers of Ter119^+^ erythroid lineage cells. Consistent with previous reports (Pui et al., 1999; Wilson et al., 2001), our analysis of Rbpj^Hem–KO^ mice revealed a remarkable reduction of thymic size and cellularity (Figure 2D,E). These studies establish that ablation of Rbpj mediated canonical notch signals in HSCs leads to defective erythroid and lymphoid differentiation.

### Lack of Rbpj Affects Radiation Stress-Induced Hematopoietic Recovery

**FIGURE 3:**
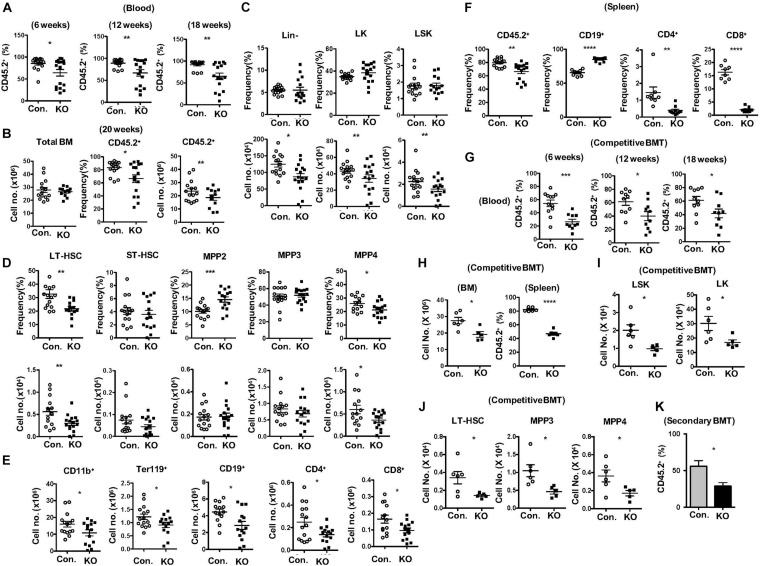
Lack of Rbpj leads to compromised radiation stress induced HSC functions. **(A)** Frequencies of donor derived chimera (CD45.2^+^) in the peripheral blood of lethally irradiated congenic recipients that received total BM of either KO or control mice at 6, 12, and 18 weeks of transplantation. **(B)** Absolute cell number of total BM (left) and frequencies (middle) and absolute cell numbers (right) of donor derived chimera (CD45.2^+^) from KO and Control recipient mice (*n* = 12–25) at 20 weeks of transplantation. **(C,D)** Frequencies (top) and absolute numbers (bottom) of Lin-, LK, LSK, LT-HSCs, ST-HSCs, MPP2, MPP3, and MPP4 subsets of BM (two femurs and two tibias) from KO and Control recipient mice (*n* = 12–15) at 20 weeks of transplantation. **(E)** Frequencies of donor derived (CD45.2^+^) CD11b^+^, Ter119^+^, CD19^+^, CD4^+^, and CD8^+^ cells in the BM (two femurs and two tibias) from KO and Control recipient mice (*n* = 10–13) at 20 weeks of transplantation. **(F)** Frequencies of donor derived (CD45.2^+^) splenocytes and CD19^+^, CD4^+^, and CD8^+^ subsets in the spleen from KO and Control recipient mice (*n* = 8–12) at 20 weeks of transplantation. **(G)** Frequencies of KO and Control derived (CD45.2^+^) chimera in the peripheral blood of lethally irradiated congenic recipients that received mixed BM of either KO or control + competitor (1:1) at 6, 12, and 18 weeks of transplantation. **(H)** CD45.2^+^KO and Control derived cell counts in the BM and frequencies in the spleen of recipients (*n* = 6) that received mixed BM of either KO or control + competitor (1:1) at 20 weeks of transplantation. **(I,J)** Absolute numbers of KO and control (CD45.2^+^) derived LK, LSK, LT-HSC, MPP3, and MPP4 subsets in the BM of recipients (*n* = 6) that received mixed BM of either KO or control + competitor (1:1) at 20 weeks of transplantation. **(K)** Frequencies of donor derived (CD45.2^+^) chimera in the peripheral blood of secondary recipients that received total BM of primary recipients from **(G)** at 8 weeks of secondary transplantation. All data represent mean ± SEM. Two-tailed Student’s *t-*tests were used to assess statistical significance (*P < 0.05; **P < 0.01; ***P < 0.001; ****P < 0.0001).

To identify functions of HSPCs in the absence of Rbpj, we performed total and mixed bone marrow transplantation (BMT) experiments. Transfer of RBC depleted total BM from Rbpj^Hem–KO^ mice into lethally irradiated wildtype (WT; CD45.1^+^) congenic recipients resulted in reduced donor (CD45.2^+^) derived hematopoiesis in the peripheral blood at 6, 12, and 18 weeks of transplantation (Figure 3A). Donor derived multi-lineage analysis indicated normal frequencies of donor myeloid and B cell, but reduced CD4^+^ and CD8^+^ T cell fractions in the peripheral blood of recipients at 18 weeks of BMT (Supplementary Figures 3A,B). Analysis of BM of these recipients, after 20 weeks of BMT, revealed a significant reduction in both absolute and relative numbers of Rbpj^Hem–KO^ donor (CD45.2^+^) derived hematopoiesis, even though the total BM cellularity of recipients was comparable between control and Rbpj^Hem–KO^ recipients (Figure 3B). To evaluate if the reduced Rbpj^Hem–KO^ donor derived hematopoiesis in irradiated hosts is caused by HSPC defects, we analyzed the HSPC pool. Our analysis identified; normal relative, but reduced absolute, numbers of Lin^–^, LK, and LSK subsets; reduced frequencies and numbers of LT-HSC and MPP4 fractions; increased relative, but normal absolute, numbers of MPP2; and normal frequencies and numbers of ST-HSC and MPP3 subsets (Figures 3C,D). Consistently, multi-lineage analysis indicated reduced absolute numbers of Rbpj^Hem–KO^ donor derived myeloid, erythroid B and T lineage cells in the BM (Figure 3E and Supplementary Figure 3C). Analysis of spleen from recipients that received Rbpj^Hem–KO^ BM suggested a reduction in overall donor derived hematopoiesis, increased frequencies of CD19^+^ B cells, and reduced frequencies of CD4^+^ and CD8^+^ T cells (Figure 3F). Next, we assessed the capacities of Rbpj mutant BM to generate hematopoiesis in the presence of WT competitor BM. Analysis of lethally irradiated recipients that received mixed BM (Rbpj^Hem–KO^: wildtype at a ratio of 1:1) indicated that the contribution of Rbpj^Hem–KO^ derived hematopoiesis was reduced at 6, 12, and 18 weeks of transplantation in the peripheral blood (Figure 3G) and at 18 weeks in BM and spleen (Figure 3H). Further analysis of Rbpj^Hem–KO^ derived HSPC compartments specified a consistent reduction of LSK, LK, LT-HSC, MPP3 and MPP4 fractions in the BM (Figures 3I,J and Supplementary Figure 3D). Finally, we transferred the BM of primary recipients from mixed chimera experiments into lethally irradiated secondary recipients and analysis indicated a remarkable reduction of Rbpj^Hem–KO^ derived hematopoiesis at 8 weeks of transplantation (Figure 3K). Overall, these data specify that Rbpj^Hem–KO^ HSPCs have reduced capacities to respond to radiation induced stress and that the HSPC defects of Rbpj^Hem–KO^ mice are caused by cell intrinsic mechanisms.

### Rbpj Deletion Causes Diminished Hematopoietic Responses to Genotoxic and Cytokine Stress

**FIGURE 4:**
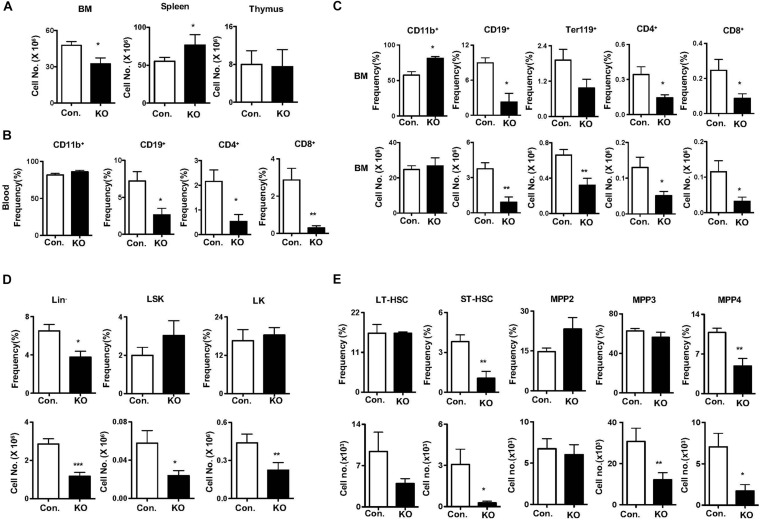
Rbpj deficiency causes defective five-Fluorouracil and cytokine induced HSC response. **(A)** Total cell counts of BM, spleen and thymus from KO and control mice after 14 days of five-FU injection (*n* = 5–7). **(B)** Frequencies of CD11b^+^, CD19^+^, CD4^+^, and CD8^+^ cells in the peripheral blood of KO and control mice after 14 days of five-FU injection (*n* = 5–7). **(C)** Frequencies (top) and absolute numbers (bottom) of CD11b^+^, CD19^+^, Ter119, CD4^+^, and CD8^+^ cells in the BM of KO and control mice after 14 days of five-FU injection (*n* = 5–7). **(D,E)** Frequencies (top) and absolute numbers (bottom) of Lin^–^, LK, LSK, LT-HSCs, ST-HSCs, MPP2, MPP3, and MPP4 subsets of BM from KO and control mice after 14 days of five-FU injection (*n* = 5–7). All data represent mean ± SEM. Two-tailed Student’s *t-*tests were used to assess statistical significance (*P < 0.05; **P < 0.01; ***P < 0.001).

To measure the responses of Rbpj deficient HSPCs to hematopoietic stress mediated by 5-Fluorouracil (5-FU) (Van Zant, 1984), we injected *i. p.* a single dose of 5-FU and hematopoietic compartments were analyzed after 14 days. Analysis of hematopoietic organs of 5-FU injected Rbpj^Hem–KO^ mice revealed reduced cellularity of the BM, increased cellularity of the spleen and normal cellularity of the thymus (Figure 4A). Multi-lineage analysis of peripheral blood indicated normal frequencies of CD11b^+^ myeloid cells, but decreased frequencies of CD19^+^ B cells, CD4^+^ T cells and CD8^+^ T cells (Figure 4B) and of BM documented increased relative, but normal absolute, numbers of myeloid, reduced relative and absolute numbers of erythroid-, B-, and T- lineage cells (Figure 4C) in 5-FU injected Rbpj^Hem–KO^ mice. Multi-lineage analysis of spleen from 5-FU injected Rbpj^Hem–KO^ mice revealed; normal numbers of myeloid cells and CD4^+^ T cells; normal relative, but increased absolute, numbers of CD19^+^ cells; and reduced relative, but normal absolute, numbers of CD8^+^ T cells (Supplementary Figures 4A,B). Finally, immunophenotyping of BM HSPC compartments from 5-FU injected Rbpj^Hem–KO^ mice identified reduced relative and absolute numbers of Lin^–^ cells and normal relative, but reduced absolute, numbers of LSK and LK cells (Figure 4D). Further characterization of LSK compartment indicated; normal relative and absolute numbers of LT-HSC and MPP2 fractions; reduced relative and absolute numbers of ST-HSCs; normal frequencies, but reduced absolute numbers, of MPP3 fraction; and reduced relative and absolute numbers of MPP4 fraction in the BM of 5-FU treated Rbpj^Hem–KO^ mice (Figure 4E).

To evaluate the capacities of Rbpj deficient HSPCs to respond to cytokine induced stress (Zhao and Baltimore, 2015) we cultured BM lin^–^ cells in the presence of HSPC cytokine cocktail (IL3 + SCF + TPO + IL6 + Flt3L). Immunophenotyping analysis indicated an altered frequencies of HSPCs in the absence of Rbpj (Supplementary Figures 4C,D). Together, these results demonstrate that the hematopoietic responses to 5-FU and cytokine-induced induced stress are compromised in the absence of Rbpj mediated notch signals.

### Disruption of Rbpj Mediated Signals Leads to Increased HSC Quiescence and p57^Kip2^ Levels

**FIGURE 5:**
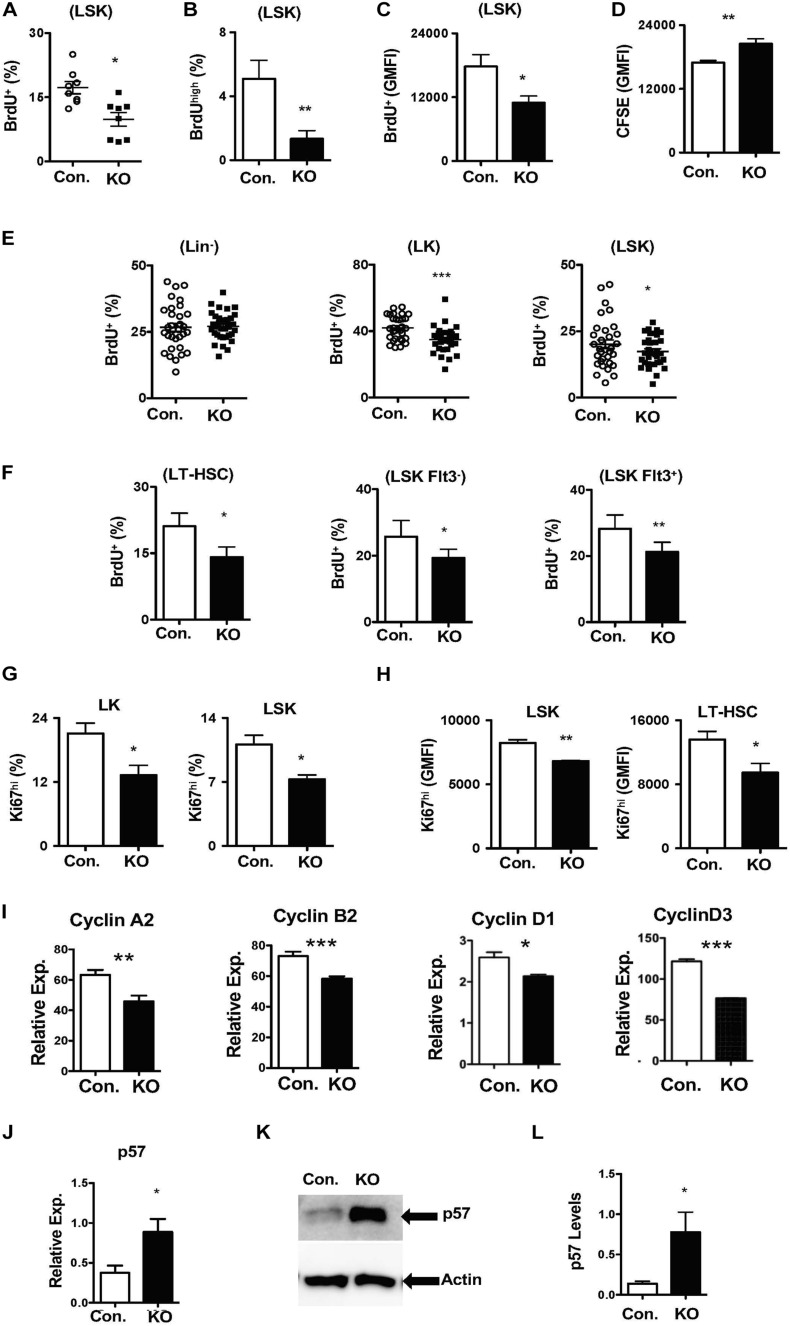
Loss of Rbpj results in increased HSC quiescence. **(A,B)** Frequencies of BrdU^+^ **(A)** and BrdU^high^ **(B)** LSK cells following *in vitro* culture of purified Lin^–^ BM cells from KO and Control mice in the presence of HSPC cytokine cocktail for 24 h. Data are pool of two independent experiments (*n* = 8–12). **(C)** Geomean fluorescence Intensity (GMFI) of BrdU^+^ LSK cells following *in vitro* culture of purified Lin^–^ BM cells from KO and Control mice in the presence of HSPC cytokine cocktail for 24 h. Data are pool of two independent experiments (*n* = 8–12). **(D)** GMFI of CFSE in LSK cells following *in vitro* culture of purified Lin^–^ BM cells from KO and Control mice in the presence of HSPC cytokine cocktail for 72 h. Data are pool of two independent experiments (*n* = 5–7). **(E,F)** Frequencies of BrdU^+^ Lin^–^, LK, LSK, LT-HSCs, Flt3^–^LSK and Flt3^+^LSK subsets in the BM of KO and Control mice (*n* = 20–22). Mice were injected i.p., with BrdU and analyzed after 24 h. **(G,H)** Frequencies of Ki67^+^ LK and LSK cells **(G)** and GMFI of Ki67^high^ LSK cells and LT-HSCs **(H)** in the BM of KO and Control mice (*n* = 10–12). **(I)** Real time PCR data for *Cyclin A2, Cyclin B2, Cyclin D1*, and *Cyclin D3* expression levels in Lin^–^ cells from the BM of KO and Control mice. Expression levels of target genes were normalized to HPRT levels. Data are representative of two independent experiments. **(J)** Real time PCR data for *p57*^*Kip2*^ expression levels in Lin^–^ cells from the BM of KO and Control mice. Expression levels were normalized to HPRT levels. Data are representative of three independent experiments. **(K)** Western blot analysis of p57^Kip2^ protein in Lin^–^ BM cells of KO and control mice. Data are representative of three independent experiments. **(L)** Quantification of proteins from western blots shown in **(K)** p57^Kip2^ protein levels were normalized to actin protein levels in each lane, respectively. Data are pool of three independent experiments. All data represent mean ± SEM. Two-tailed Student’s *t-*tests were used to assess statistical significance (*P < 0.05; **P < 0.01; ***P < 0.001).

To identify the cellular mechanisms responsible for diminished stress-induced hematopoietic response, we focused on the proliferation kinetics of HSPCs. Indeed, capacities of HSCs to exit quiescence and enter an active proliferative state is vital for the demand-adapted regulation of hematopoiesis under severe stress conditions (Takizawa et al., 2012). First, we assessed the *in vitro* proliferative capacities of Rbpj deficient HSPCs through BrdU pulsing studies. Data indicated that the frequencies of BrdU^+^ (Figure 5A) and BrdU^high^ (Figure 5B) Rbpj mutant LSK cells were reduced, even though their viability rates were normal (Supplementary Figure 5A). Further quantitative analysis suggested a reduction in the amount of incorporated BrdU within BrdU^+^ Rbpj mutant LSK cells (Figure 5C), but not in Lin^–^ and LK cells (Supplementary Figures 5B,C). To further verify these data, through an independent approach, we performed CFSE dilution assays and data confirmed that Rbpj mutant LSK cells exhibit reduced proliferation capacities *in vitro* in the presence of HSPC cytokines (Figure 5D).

Based on these data, we hypothesized that loss of Rbpj leads to decreased proliferative responses in HSPCs. To validate this and further strengthen our findings, we performed short-term *in vivo* BrdU pulsing experiments. Rbpj^Hem–KO^ mice were injected *i.p* with BrdU and BM HSPCs were analyzed after 24 h. Whereas the proliferation rates were normal in Lin^–^ cells, the frequencies of proliferating (BrdU^+^) LSK and LK cells were reduced in the BM of Rbpj^Hem–KO^ mice (Figure 5E). Further analysis specified decreased proliferation of LT-HSCs (CD150^+^CD48^–^LSK), Flt3^–^LSK, and Flt3^+^ LSK cells from Rbpj^Hem–KO^ mice (Figure 5F). To corroborate these findings, we quantified Ki67 levels, a faithful marker of cell proliferation, in Rbpj^Hem–KO^ HSPCs. Data indicated that the frequencies of Ki67^+^ LK, and LSK cells (Figure 5G and Supplementary Figure 5D) and expression levels of Ki67 within Ki67^+^ LSK cells and LT-HSCs (Figure 5H and Supplementary Figure 5E) were reduced.

To explain these findings at a molecular level, we quantified expression levels of both positive (Cyclins) and negative [cyclin dependent kinase inhibitors (CDKIs)] regulators of cell cycle in Rbpj deficient Lin- BM cells. Real-time PCR assays documented reduced mRNA levels of key cyclins, such as *CyclinA2*, *CyclinB2*, *CyclinD1*, and *CyclinD3* (Figure 5I). On the other hand, mRNA and protein expression levels of *p57*^*Kip2*^, a key CDKI and positive regulator of HSC quiescence (Matsumoto et al., 2011; Zou et al., 2011), were augmented in the absence of Rbpj (Figures 5J–L). However, mRNA levels of other CDKIs, including *p16*, *p18*, and *p27*, were reduced in Rbpj mutant cells Lin- BM cells (Supplementary Figure 5F). Taken together, these studies suggest that Rbpj mediated signaling pathways are essential for proper transition of HSCs from a quiescent state to proliferative state and finetuning the balance between cyclins and CDKIs.

### Loss of Rbpj Causes Augmented Expression Notch Target Genes and Hes1 Functions

**FIGURE 6:**
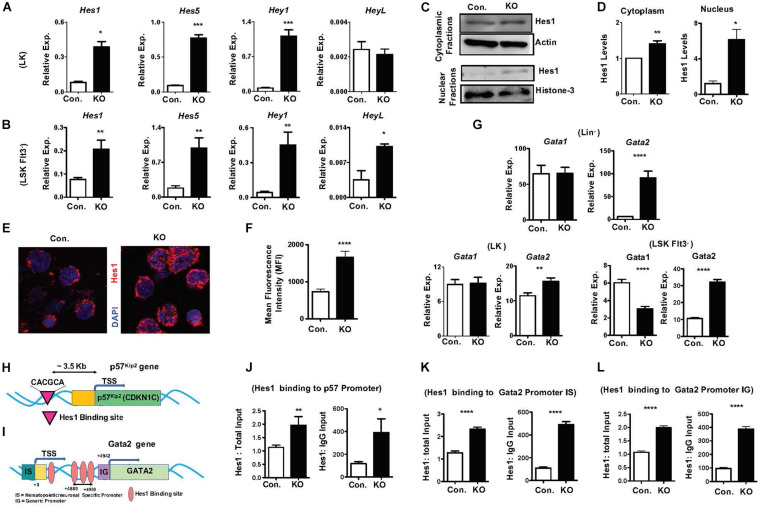
Lack of Rbpj leads to augmented expression and activity of notch target genes. **(A,B)** Real time PCR analysis of notch target genes in purified LK **(A)** and Flt3^–^LSK **(B)** cells of BM from KO and control mice. Expression levels were normalized to HPRT levels. Data are representative of three independent experiments. **(C)** Western blot analysis of Hes1 proteins in the cytoplasm and nucleus of Lin^–^ BM cells of KO and control mice. Data are representative of three independent experiments. **(D)** Quantification of proteins from western blots shown in **(C)** cytoplasmic Hes1 protein levels were normalized to actin protein levels and nuclear Hes1 protein levels were normalized to Histone-3 protein levels in each lane, respectively. Data are pool of three independent experiments. **(E)** Representative immunofluorescence images of Hes1 and DAPI in purified Lin^–^c-Kit^+^ BM cells of KO and control mice. **(F)** Quantification of nuclear Hes1 levels from **(E)**. Data points include *n* = 30–40 individual cells from two independent experiments. **(G)** Real time PCR analysis of *Gata1* and *Gata2* mRNA levels in purified Lin^–^, LK and Flt3^–^LSK cells of BM from KO and control mice. Expression levels were normalized to HPRT levels. Data are representative of two independent experiments. **(H)** Diagrammatic representation of *p57*^*Kip2*^ gene indicating the presence of Hes1 binding site in the 5′ upstream of its promoter. **(I)** Diagrammatic representation of *Gata2* gene indicating the presence of four Hes1 binding sites in both hematopoietic/neuronal specific promoter (IS) and generic promoter (IG) regions of *Gata2.* **(J)** ChIP analysis of Hes1 binding to the regulatory regions of *p57*^*Kip2*^ in purified Lin^–^ cells of BM from KO and control mice. Shown are the real-time PCR data of Hes1 immunoprecipitates, which were normalized to either total input (left) or IgG control (right) immunoprecipitates. Data are representative of two independent experiments. **(K,L)** ChIP analysis of Hes1 binding to the IS **(K)** and IG **(L)** promoter regions of *Gata2* in purified Lin^–^ cells of BM from KO and control mice. Shown are the real-time PCR data of Hes1 immunoprecipitates, which were normalized to either total input (left) or IgG control (right) immunoprecipitates. Data are representative of two independent experiments. All data represent mean SEM. Two-tailed Student’s *t*-tests were used to assess statistical significance (^∗^*P* < 0.05; ^∗∗^*P* < 0.01; ^∗∗∗^*P* < 0.001; ^****^P < 0.0001).

To identify molecular mechanisms responsible for the HSPC phenotype of Rbpj^Hem–KO^ mice, we assessed the expression levels of notch target genes in the absence of Rbpj. In view of the fact that Rbpj is the key downstream mediator and indispensable for the canonical notch pathway (Kopan and Ilagan, 2009; Pajcini et al., 2011; Bigas and Espinosa, 2012; Bray, 2016), we anticipated that expression of notch targets might be reduced in Rbpj mutant HSPCs. Unexpectedly, our studies identified that the expression levels of key downstream targets of Notch pathway (Kopan and Ilagan, 2009; Pajcini et al., 2011; Bigas and Espinosa, 2012; Bray, 2016), including *Hes1*, *Hes5*, *Hey1*, and *HeyL*, were elevated in Rbpj mutant HSPCs (Figures 6A,B). We were especially intrigued by the elevated expression of *Hes1* in Rbpj deficient HSPCs, as Hes1 has been considered as the key and major target gene of canonical Notch pathway in hematopoietic cells (Gordon et al., 2008; Kopan and Ilagan, 2009; Radtke et al., 2010; Pajcini et al., 2011; Lobry et al., 2014). To strengthen these results, we quantified the expression levels of Hes1 protein. Our western blot studies concluded that Hes1 protein levels were elevated in both cytoplasm and nucleus of Rbpj deficient hematopoietic progenitors (Figures 6C,D). To further corroborate these data, we performed confocal microscopy studies. Consistent with our observations, a remarkably increased levels of Hes1 protein were detected in the nucleus of Rbpj mutant HSPCs (Figures 6E,F).

All data represent mean ± SEM. Two-tailed Student’s *t-*tests were used to assess statistical significance (^∗^*P* < 0.05; ^∗∗^*P* < 0.01; ^∗∗∗^*P* < 0.001; ^****^P < 0.0001).

To identify the functional consequences of elevated *Hes1*, we analyzed if expression of transcription factors, such as *Gata1, Gata2, c-Myc, HoxA9, Cebp*α, and *Gfi1*, that play key roles in hematopoiesis is altered in Rbpj^Hem–KO^ HSPCs. These studies identified normal expression of *Gata1* and *c-Myc* and increased expression of *Gata2* and *Gfi1* in Lin^–^ cells, normal expression of *Gata1* and *Cebp*α and increased expression of *Gata2* and *HoxA9* in LK cells, and reduced expression of *Gata1*, increased expression of *Gata2* and normal expression of *HoxA9* and *Cebp*α in LSKFlt3^–^ cells (Figure 6G and Supplementary Figures 6A–C). Among these candidate transcription factors, we were intrigued by deregulated expression of Gata2 because; (1) Gata2 is consistently upregulated in Lin^–^, LK and LSKFlt3^–^ cells of Rbpj^Hem–KO^ mice; (2) overexpression of Gata2 in mouse and human HSCs (Tipping et al., 2009; Nandakumar et al., 2015) has been shown to block hematopoietic reconstitution in irradiated hosts, inhibit cell cycle entry/promote quiescence and suppress lymphoid differentiation, very similar to the phenotype of Rbpj deficient HSPCs; and (3) Hes1 has been suggested to regulate *Gata2* expression during embryogenesis (Guiu et al., 2013).

To test if increased expression levels of *Gata2* (Figure 6G) and p57^Kip2^ (Figures 5J–L) in Rbpj mutant HSPCs are in response to augmented binding of Hes1 to their promoters, we performed *in silico* analysis. Indeed, a single Hes1 binding site (CACGCA) is present in *p57*^*Kip2*^ promoter (Figure 6H) and four Hes1 binding sites (Guiu et al., 2013) in the regulatory regions of both generic and hematopoietic specific promoters of *Gata2* (Figure 6I). Finally, data of Chromatin immunoprecipitation (ChIP) experiments documented an increased binding of Hes1 to the promoters of *p57*^*Kip2*^ and *Gata2* in Lin- BM cells of Rbpj^Hem–KO^ mice (Figures 6J–L). Overall, these molecular studies established that Rbpj deficiency causes elevated expression of notch target genes and increased binding of Hes1 to the regulatory regions of *p57*^*Kip2*^ and *Gata2.*

### Loss of Rbpj Induces Hif1α Mediated Upregulation of Hes1

**FIGURE 7:**
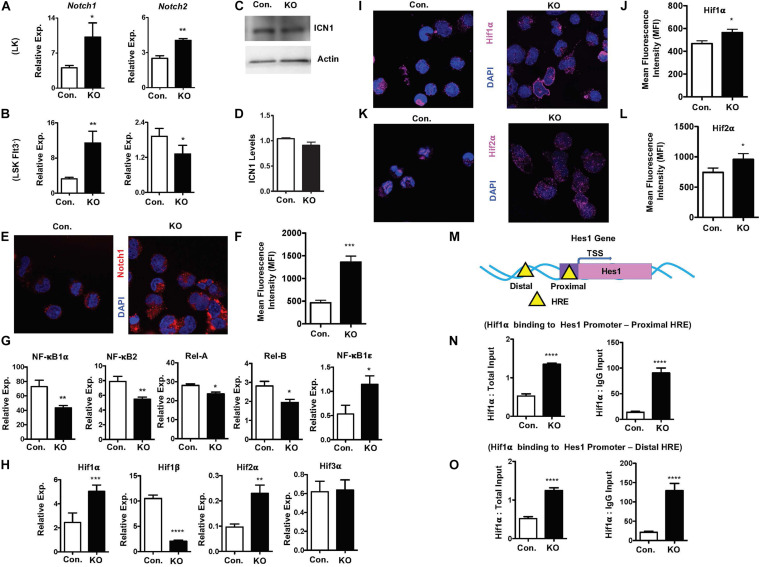
Rbpj loss results in elevated Hif expression and functions. **(A,B)** Real time PCR analysis of *notch1* and *notch2* mRNA expression in purified LK **(A)** and Flt3^–^LSK **(B)** cells of the BM from KO and control mice. Expression levels were normalized to HPRT levels. Data are representative of three independent experiments. **(C)** Western blot analysis of Notch1 protein in Lin^–^ BM cells of KO and control mice. Data are representative of three independent experiments. **(D)** Quantification of proteins from western blots shown in **(C)**. Notch1 protein levels were normalized to actin protein levels in each lane, respectively. Data are pool of two independent experiments. **(E)** Representative immunofluorescence images of Notch1 and DAPI in purified Lin^–^ BM cells of KO and control mice. **(F)** Quantification of nuclear Notch levels from **(E)**. Data points include *n* = 30 individual cells from two independent experiments. **(G)** Real time PCR analysis of NF-κB target genes in purified Lin^–^ BM cells from KO and control mice. Expression levels were normalized to HPRT levels. Data are representative of three independent experiments. **(H)** Real time PCR analysis of *Hif1α, Hif2α, Hif3*α, and *Hif1β* target genes in purified Lin^–^ BM cells from KO and control mice. Expression levels were normalized to HPRT levels. Data are representative of three independent experiments. **(I,K)** Representative immunofluorescence images of Hif1α and DAPI **(I)** and Hif2α and DAPI **(K)** in purified Lin^–^ BM cells of KO and control mice. **(J,L)** Quantification of nuclear Hif1α levels **(J)** from **(I)** and Hif2α levels **(L)** from **K**. Data points include *n* = 60 **(J)** and *n* = 30 **(L)** individual cells from two independent experiments. **(M)** Diagrammatic representation of *Hes1* gene indicating the presence of Hif binding HRE sites in both the promoter (proximal) and 5′ upstream (distal) regulatory regions. **(N,O)** ChIP analysis of Hif1α binding to the promoter **(N)** and 5′ upstream regulatory **(O)** regions of *Hes1* in purified Lin^–^ cells of BM from KO and control mice. Shown are the real-time PCR data of Hif1α immunoprecipitates, which were normalized to either total input (left) or IgG control (right) immunoprecipitates. Data are representative of two independent experiments. All data represent mean ± SEM. Two-tailed Student’s *t-*tests were used to assess statistical significance (*P < 0.05; **P < 0.01; ***P < 0.001; ****P < 0.0001).

To investigate pathways leading to elevated expression of notch target genes, we assessed if notch activity is intact in the absence of Rbpj. Real-Time PCR assays revealed that expression levels of *Notch1* and *Notch2* mRNA were increased in LK and Flt3^–^LSK cells of Rbpj^Hem–KO^ mice (Figures 7A,B). While western blot analysis indicated normal levels of cleaved Notch1 (ICN1) protein (Figures 7C,D), confocal microscopy studies documented an augmented levels of Notch1 in the nucleus of Rbpj mutant progenitor cells (Figures 7E,F). These findings were unexpected, because it has been believed that Rbpj deficiency ablates all canonical notch signaling pathways.

To further understand and possibly explain these findings, we assessed if non-canonical notch pathways are altered in Rbpj^Hem–KO^ mice. First, we explored if NF-κB signaling is augmented in the absence of Rbpj, as signaling cross talks exist between notch and NF-κB pathways (Andersen et al., 2012; Ayaz and Osborne, 2014). Real-time PCR analysis indicated rather reduced expression levels of NF-κB target genes, such as *NF-κB1α, NF-κB2, Rela*, and *Relb*, even though expression levels of *NF-κB1ε* was elevated, in Rbpj mutant progenitor cells (Figure 7G). Next, we tested if expression levels of Hypoxia inducible factor (Hif) family members are altered in Rbpj mutant cells. Even though Hif proteins are implicated in non-canonical Notch pathways (Gustafsson et al., 2005; Johnson, 2011; Mukherjee et al., 2011; Hu et al., 2014), it remained totally unknown if Hif proteins mediated non-canonical notch pathway plays any functions in hematopoiesis, particularly in HSPCs. Our gene expression studies documented an upregulation of *Hif1*α and *Hif2*α mRNA, downregulation of *Hif1β* and normal expression of *Hif3*α mRNA in Rbpj deficient progenitor cells (Figure 7H). Consistently, confocal microscopy studies documented an increased presence of Hif1α and Hif2α proteins in the nucleus of Rbpj deficient progenitor cells (Figures 7I–L). Despite increased expression of Hif1α and Hif2α, intracellular reactive oxygen species (ROS) levels in Rbpj mutant HSPCs remain normal (Supplementary Figures 7A,B), suggesting that the increased expression Hif1*α* and Hif2*α* are not in response to hypoxia. Finally, to determine the functional consequences of elevated levels of Hif proteins, we assessed if Hes1 is a direct transcriptional target of Hif1*α* in HSPCs. *In silico* studies suggested the presence of two Hif binding (HRE) sites (Zheng et al., 2017), one in the promoter and the other in the 5′ regulatory region, of *Hes1* gene (Figure 7M). ChIP assays documented an elevated Hif1α binding to both proximal and distal Hypoxia Responsive Element (HRE) sites of *Hes1* (Figures 7N,O) in BM progenitor cells. Taken together, these studies documented that Rbpj deficiency leads to increased expression and functional activity of Hif proteins in HSPCs.

## Discussion

Functional relevance of Notch signaling in the maintenance of adult HSCs remains a controversial subject. *In vitro* studies based on overexpression of Notch receptors and treatment of HSCs with notch ligands have provided a compelling evidence on the positive role of notch in HSCs (Pui et al., 1999; Varnum-Finney et al., 2000; Stier et al., 2002; Duncan et al., 2005; Rathinam et al., 2006; Francis et al., 2017). On the other hand, studies mainly based on the loss-of-functions approach have concluded that notch signaling is dispensable for the maintenance and functions of adult HSCs (Mancini et al., 2005; Maillard et al., 2008; Varnum-Finney et al., 2011; Benveniste et al., 2014; Francis et al., 2017). Even though there appears to be an obvious disagreement regarding the functions of notch in HSCs, these differences are likely multifactorial and possible explanations for these discrepancies could be as follows; (1) In view of the fact that are multiple notch receptors and their ligands in the mammalian system, it remains possible that deletion of an individual notch receptor/ligand can be compensated by the other notch receptors. Indeed, multiple notch receptors; Notch1 (Varnum-Finney et al., 2000; Rathinam et al., 2006), Notch2 (Varnum-Finney et al., 2011; Wang et al., 2017) and Notch4 (Vercauteren and Sutherland, 2004) have been shown to play roles in HSCs and hematopoiesis, (2) To overcome the possible functional redundancies amongst notch receptors/ligand, studies were conducted based on global ablation of notch signals, through either retroviral mediated overexpression/genetic activation of dnMAML (Maillard et al., 2008; Benveniste et al., 2014; Francis et al., 2017) or genetic ablation of Rbpj (Maillard et al., 2008). Data of these studies have suggested a dispensable role for notch in HSCs. While inhibiting MAML and Rbpj can efficiently block canonical notch pathways, mounting evidences suggest an existence of Rbpj independent functions of notch pathway (Turkoz et al., 2016), and (3) Notch signaling is controlled at multiple levels and through various mechanisms. Even though deletion of individual notch receptors did not affect adult HSC maintenance or functions, conditional loss of O-fucosylglycans on Notch EGF-like repeats, which results in defective binding of all Notch ligands, in adult HSCs caused reduced pool size, loss of quiescence, altered niche distribution and increased mobilization of HSPCs (Wang et al., 2017). In the present study we document that loss of canonical notch pathway leads to reduced capacities of HSCs to differentiate into lymphoid-primed multipotent progenitors. Even though both long-term and short-term HSC pool was largely normal in Rbpj mutant mice under steady state conditions, their functions are severely compromised in response to radiation and chemotherapy induced hematopoietic stress. Interestingly, earlier studies by Duarte et al. (2018) concluded that canonical notch signals are dispensable for adult steady-state and stress myelo-erythropoiesis. In contrast, data obtained from our studies unequivocally demonstrate that Rbpj mediated canonical notch signaling is critical for stress-induced hematopoiesis. Even though the conclusions of our studies and of Daurte *et al.* were based on Rbpj conditional KO mice, the Cre-deleter strains used in these two studies are completely different. Daurte et al. conducted most of their studies, including their BM transplantation experiments, using Mx1-cre deleter strain. However, it has become apparent in the recent years that Mx1-cre based deletion of transgenes has some potential pitfalls (Velasco-Hernandez et al., 2016). More importantly, in view of the fact that injection of poly I:C, necessary to induce expression of Cre in Mx1-Cre mouse strain, activates the interferon system and that interferons alter hematopoiesis and HSC physiology (Essers et al., 2009; Passegue and Ernst, 2009), it is unclear to what extent the studies and conclusion of Daurte et al. were influenced by the interferon mediated “effects”. To overcome these technical hurdles, in the present study we genetically ablated Rbpj signals using Vav-cre deleter strain and this strategy allowed us to constitutively ablate canonical notch signals in all hematopoietic cells from embryo throughout adulthood. Moreover, Duarte et al. (2018) particularly focused on stress erythropoiesis, mediated by Phenyl-hydrazine (PHZ), and concluded that Rbpj has a dispensable role in their model. In contrast, we studied global stress responses of HSCs, induced by 5-Fluorouracil (5-FU), and our data identified that Rbpj has an indispensable role in HSC mediated stress response. Taken together, these studies warrant a need for careful interpretation of data on notch functions in HSCs and for thorough research, involving additional and more sophisticated models to ablate global notch signals.

Mounting evidences document the existence of RBPJ independent non-canonical Notch signaling pathways (Andersen et al., 2012; Ayaz and Osborne, 2014). Intriguingly, most often non-canonical notch signals are associated with pathological conditions, including myeloproliferative disorders (Wang et al., 2014) human myeloid leukemia (Liao et al., 2007) and T cell leukemia (Ayaz and Osborne, 2014). While these studies established the significance of non-canonical notch signals in pathophysiology, to date nothing is known about their roles and downstream molecular consequences in HSCs. Our data on Rbpj deficient mice, for the first time, suggest that loss of canonical notch pathway in HSPCs, leads to activation of Hif1α mediated regulation of notch targets. Earlier studies documented an interesting role for Hifα proteins in the activation of non-canonical notch pathways. According to our current understanding, Hif proteins physically interact with notch receptors and colocalized in endocytic vesicles (Johnson, 2011; Mukherjee et al., 2011). This interaction causes ligand independent cleavage of ICD of notch from the cell membrane and stabilization of Hifα proteins (Johnson, 2011; Mukherjee et al., 2011) and causes transcriptional activation of notch target genes in the nucleus, including *Hes1* (Gustafsson et al., 2005; Pistollato et al., 2010; Zheng et al., 2017). Non-canonical notch activation by Hifα has been reported to exist in cells under both normoxic and hypoxic conditions, and pathological states (Gustafsson et al., 2005; Pistollato et al., 2010; Hu et al., 2014; Zheng et al., 2017). Even though Rbpj has been identified as the key and major component of the notch induced transcriptional activation complex, Hifα/notch mediated transcriptional activation of notch target genes is independent of Rbpj (Gustafsson et al., 2005; Pistollato et al., 2010; Hu et al., 2014; Zheng et al., 2017). Furthermore, earlier studies identified that Rbpj physically interacts with Hif proteins and suppresses Hif activity under steady state conditions (Diaz-Trelles et al., 2016). Consistent with these observations, data from our current study document that Rbpj deficiency causes an augmented Hifα activity in hematopoietic progenitor cells. Based on these studies, and our current findings, it is tempting to hypothesize that loss of Rbpj leads to defective suppression of Hif activity, which in results in exaggerated non-canonical activation of notch pathways in hematopoietic cells. We believe that exaggerated Hif activity may compensate for the loss of canonical notch signals under steady state conditions. However, this compensatory role by Hif1α is insufficient for initiating effective stress induced-hematopoietic recovery. In addition, it is currently unclear to what extent Hif1α compensates for the loss of Rbpj signals. Even though more studies are necessary for understanding the molecular interactions and targets of Hif1α in these settings, our data document the existence and highlight the functions of Hif1α mediated non-canonical notch signaling pathways in HSCs.

Even though our mechanistic studies provide novel insights and rationale as how and why loss of canonical notch signals might result in normal hematopoiesis (under steady state hematopoiesis), we believe that additional mechanisms might be involved in the phenotype of Rbpj mutant HSCs. More thorough investigations, particularly, focused on Rbpj/Notch dependent and independent (but Hif1α dependent) activation of Hes1 might be necessary for understanding the complex roles played by notch signaling pathways in HSPCs.

## Data Availability Statement

The raw data supporting the conclusions of this article will be made available by the authors, without undue reservation.

## Ethics Statement

The animal study was reviewed and approved by the Institutional Animal Care and Use Committee.

## Author Contributions

RL performed experiments, collected the data, and analyzed the data. CR designed and performed research, analyzed and interpreted the data, and wrote and corrected the manuscript. Both authors contributed to the article and approved the submitted version.

## Conflict of Interest

The authors declare that the research was conducted in the absence of any commercial or financial relationships that could be construed as a potential conflict of interest. The reviewer, MN, declared a past co-authorship with one of the authors, CR, to the handling editor.
