# Supplementary material for: Deficiency of Rbpj Leads to Defective Stress-Induced Hematopoietic Stem Cell Functions and Hif Mediated Activation of Non-canonical Notch Signaling Pathways
Source: Front Cell Dev Biol. 2021 Jan 25;8:622190. doi: 10.3389/fcell.2020.622190 (PMC7868433; doi:10.3389/fcell.2020.622190)
Supplement: Supplementary file 1 [file Data_Sheet_1.PDF]

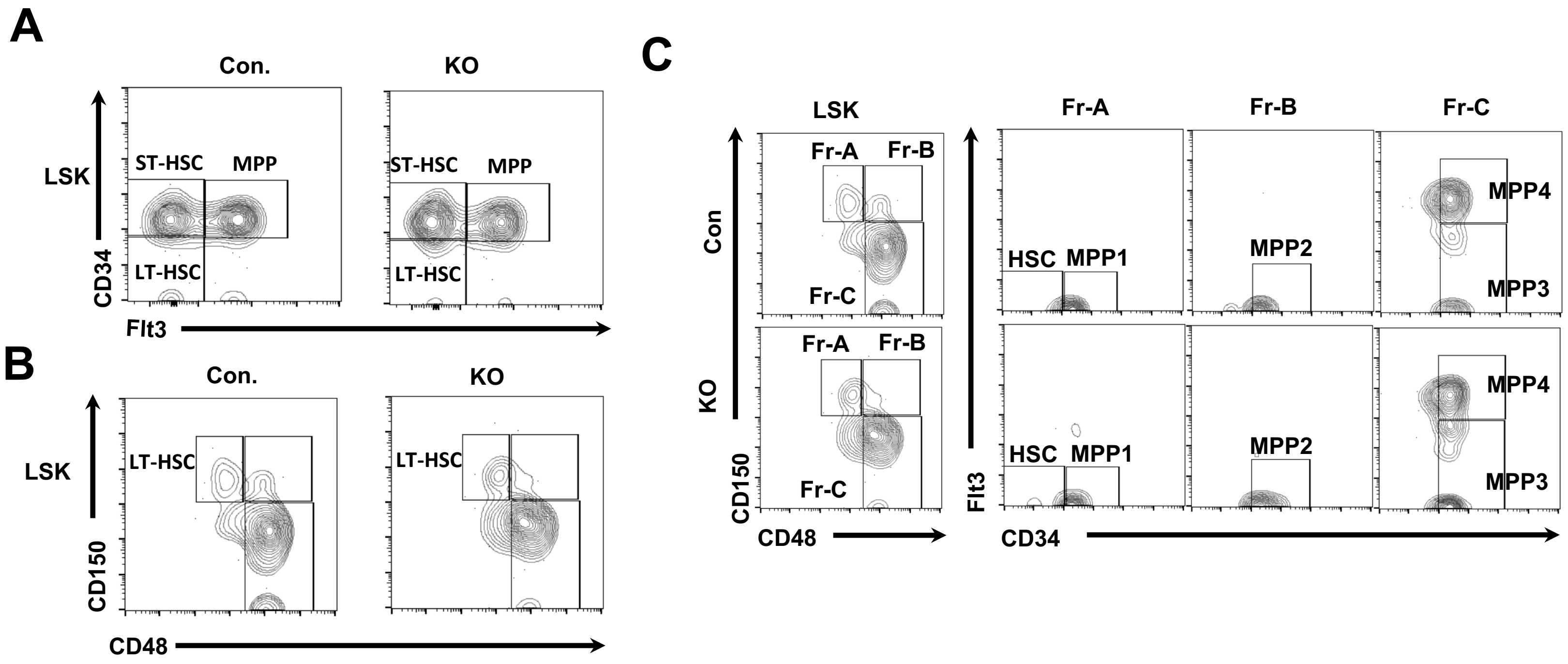
A
Con.
KO
LSK
ST-HSC
MPP
LT-HSC
CD34
Flt3
B
Con.
KO
LSK
LT-HSC
CD150
CD48
C
LSK
Fr-A
Fr-B
Fr-C
Con
KO
CD150
CD48
Fr-A
Fr-B
Fr-C
HSC
MPP1
MPP2
MPP3
MPP4
Flt3
CD34

Figure S1.

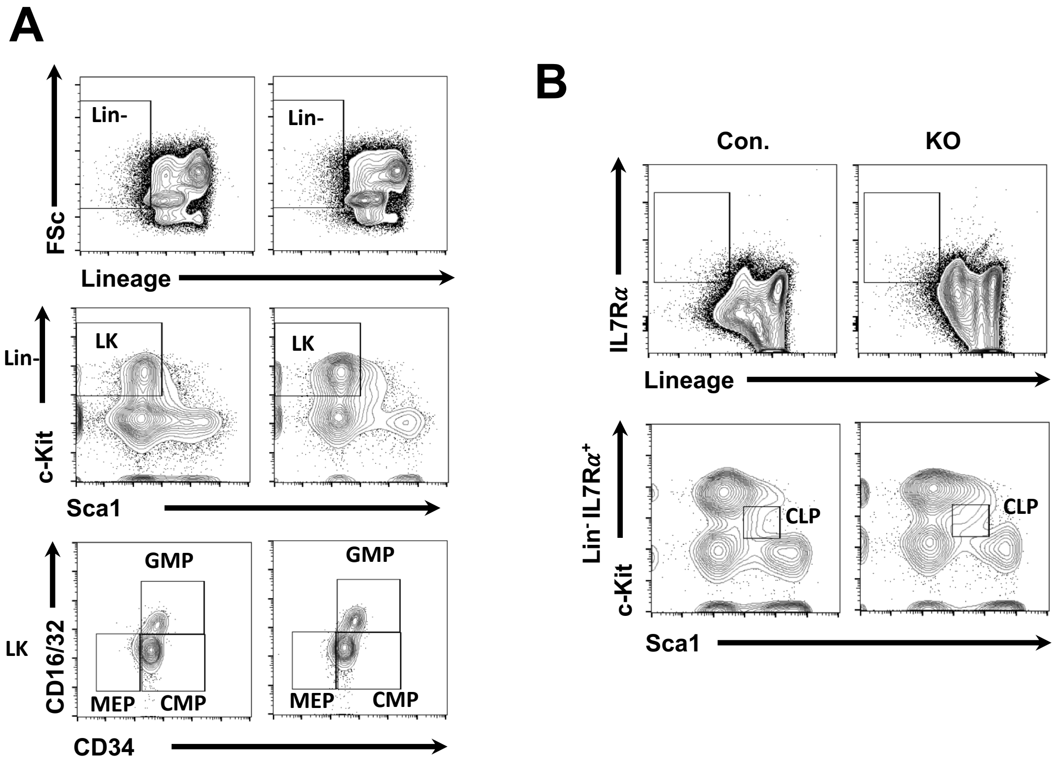
A
Lin-
Lin-
FSc
Lineage
LK
LK
Lin-
c-Kit
Sca1
GMP
GMP
LK
CD16/32
MEP
CMP
MEP
CMP
CD34
B
Con.
KO
IL7Rα
Lineage
CLP
CLP
Lin⁻IL7Rα⁺
c-Kit
Sca1

Figure S2.

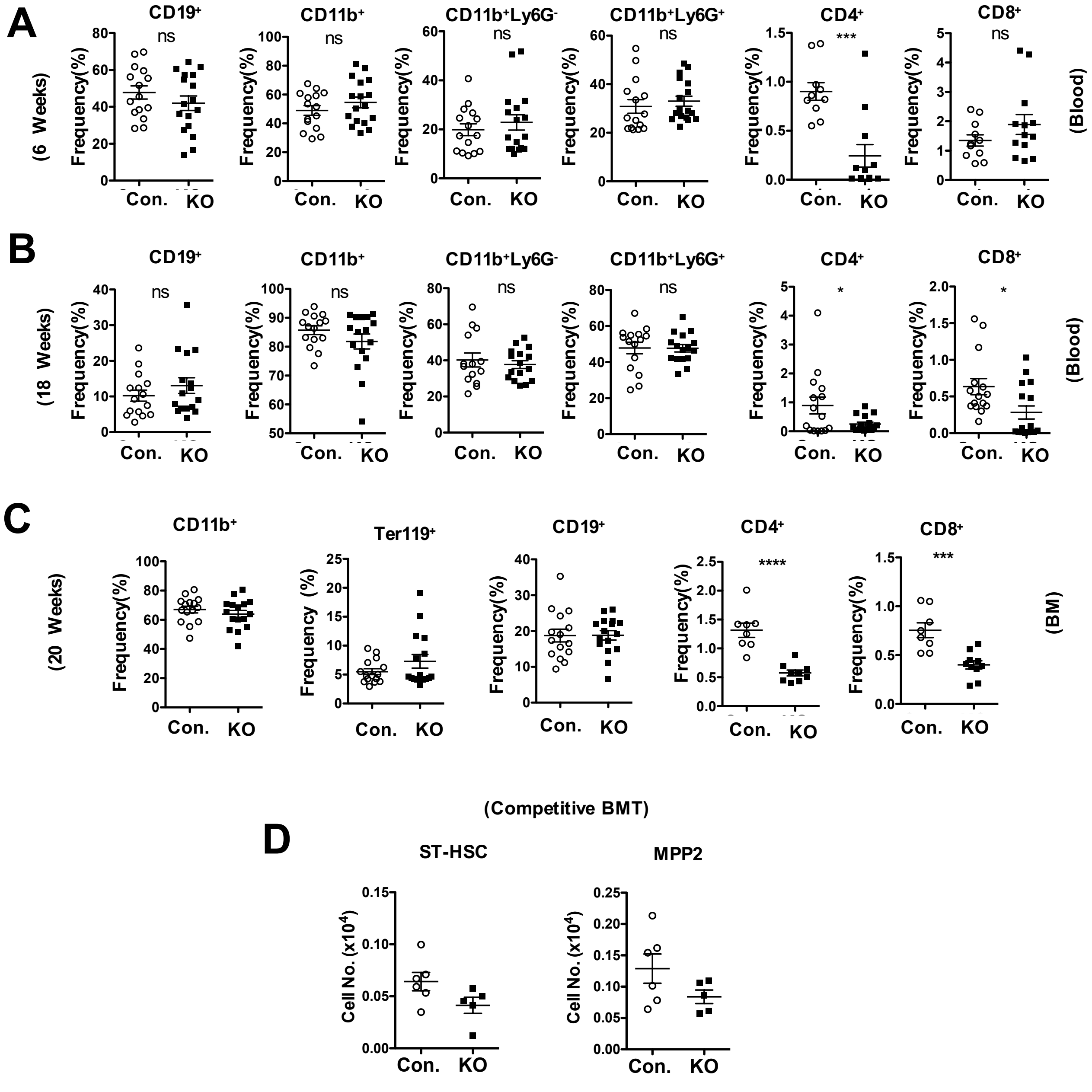
A
CD19+
CD11b+
CD11b+Ly6G-
CD11b+Ly6G+
CD4+
CD8+
(6 Weeks)
(Blood)
Frequency(%)
ns
***
Con.
KO
B
(18 Weeks)
*
C
(20 Weeks)
(BM)
Ter119+
Frequency (%)
****
(Competitive BMT)
D
ST-HSC
MPP2
Cell No. (x10^4)

Figure S3.

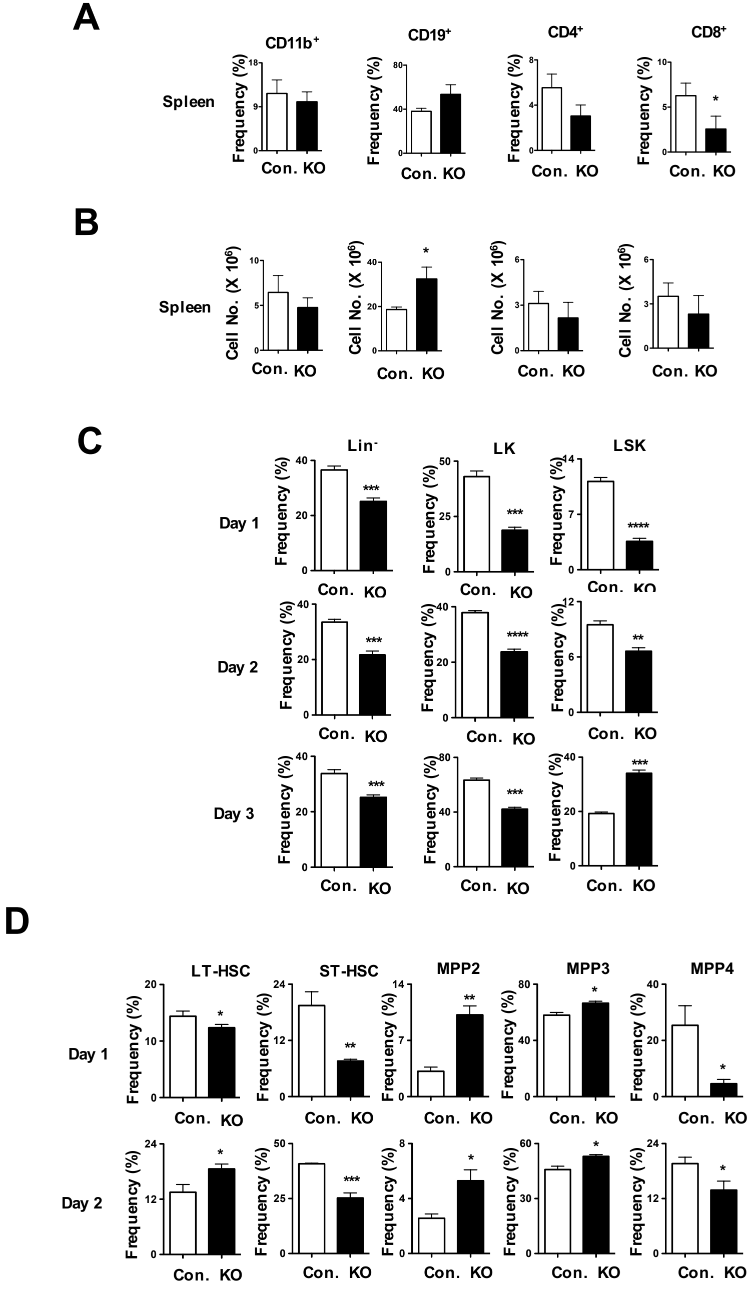
A
CD11b+
CD19+
CD4+
CD8+
Spleen
Frequency (%)
Con. KO
B
Spleen
Cell No. (X 10^6)
Con. KO
C
Lin-
LK
LSK
Day 1
Day 2
Day 3
Frequency (%)
Con. KO
D
LT-HSC
ST-HSC
MPP2
MPP3
MPP4
Day 1
Day 2
Frequency (%)
Con. KO

Figure S4.

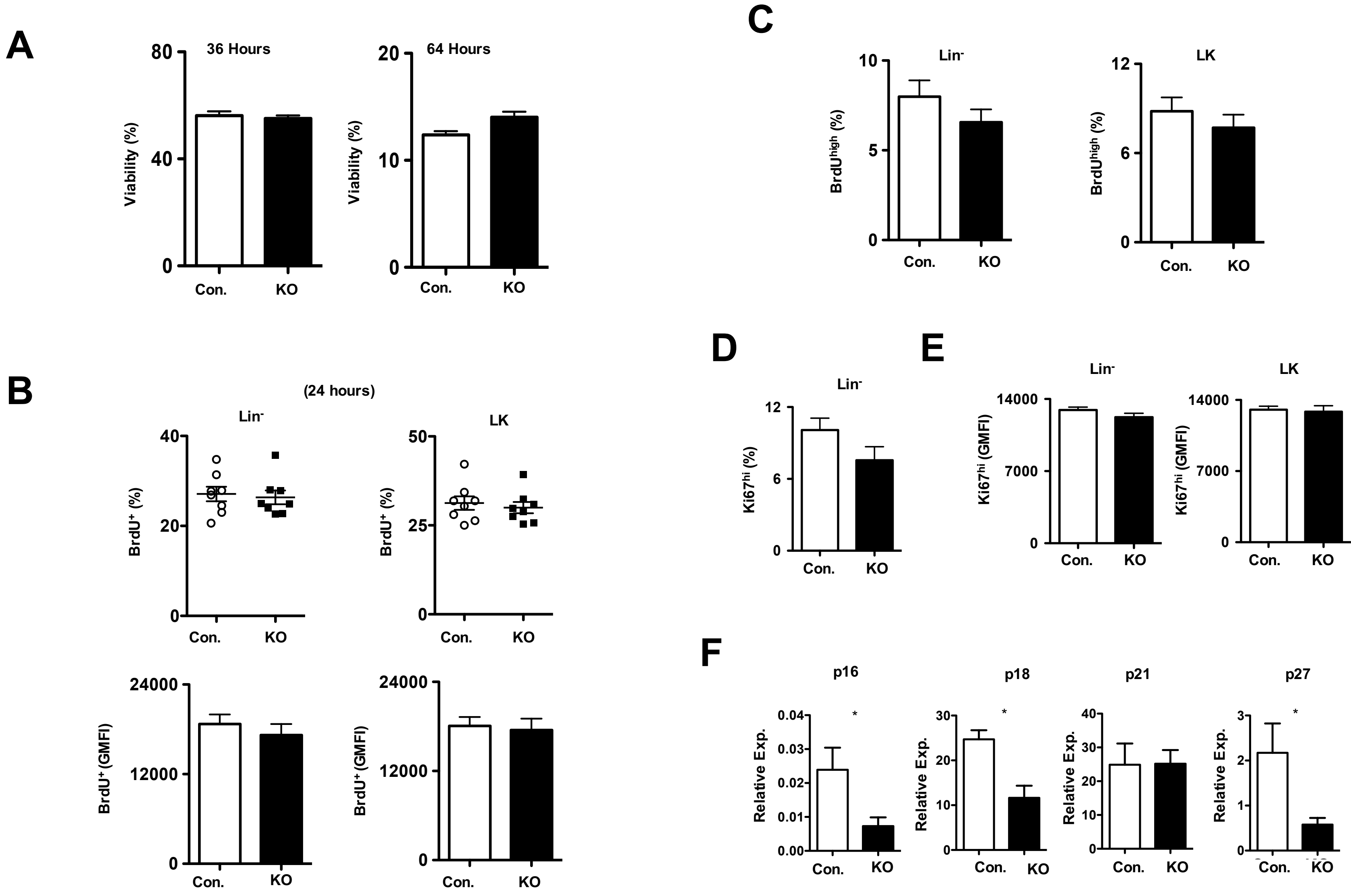
A
36 Hours
64 Hours
Viability (%)
Con.
KO
B
(24 hours)
Lin-
LK
BrdU+ (%)
BrdU+ (GMFI)
C
BrdUhigh (%)
D
Ki67hi (%)
E
Ki67hi (GMFI)
F
p16
p18
p21
p27
Relative Exp.

Figure. S5

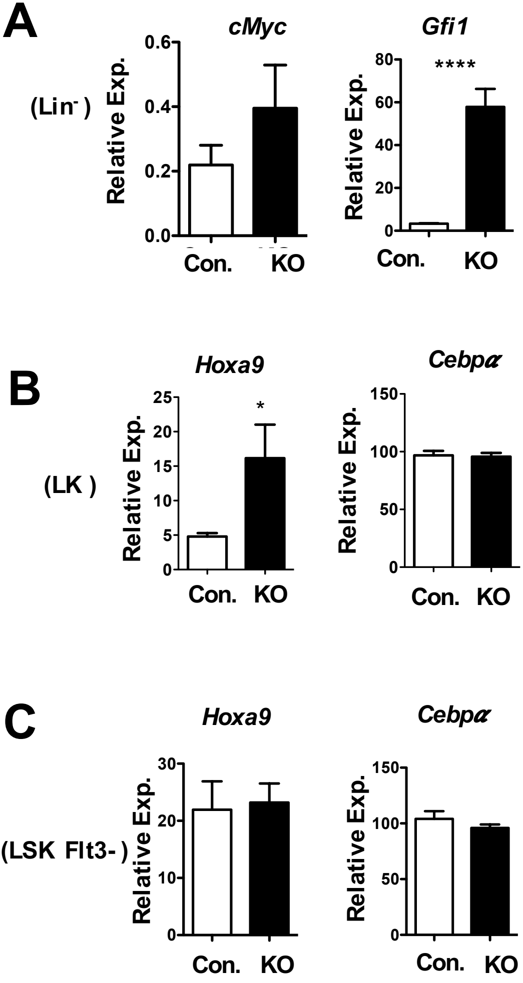
A
(Lin-)
cMyc
Relative Exp.
0.6
0.4
0.2
0.0
Con.
KO
Gfi1
****
80
60
40
20
0
B
(LK)
Hoxa9
*
25
20
15
10
5
Cebpα
150
100
50
C
(LSK Flt3-)
30

Figure. S6

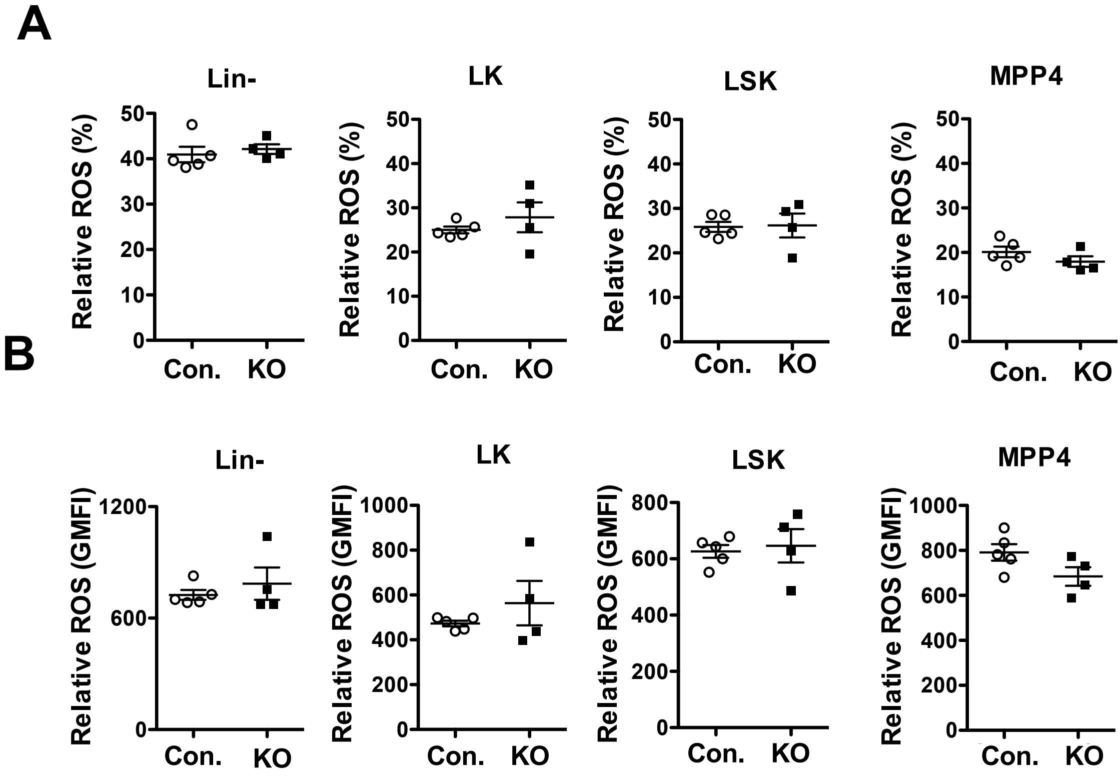
A
Lin-
LK
LSK
MPP4
Relative ROS (%)
0
10
20
30
40
50
Con.
KO
B
Relative ROS (GMFI)
0
600
1200
200
400
600
800
1000
Con.
KO

Figure S7.

Supplemental Figure Legends

Figure S1.

A. FACS plots indicating gating strategy used for LT-HSC ($CD34^-Flt3^-$), ST-HSC ($CD34^+Flt3^-$) and MPP ($CD34^+Flt3^+$) analysis of BM (two femurs and two tibias) from KO and control mice. Data are representative of 7 independent experiments.

B. FACS plots indicating gating strategy used for LT-HSC ($CD150^+CD48^-$) analysis of BM (two femurs and two tibias) from KO and control mice. Data are representative of 7 independent experiments.

C. FACS plots indicating gating strategy used for HSC, MPP1, MPP2, MPP3 and MPP4 analysis of BM (two femurs and two tibias) from KO and control mice. Data are representative of 7 independent experiments.

Figure S2.

A. FACS plots indicating gating strategy used for CMP, GMP and MEP analysis of BM (two femurs and two tibias) from KO and control mice. Data are representative of 3 independent experiments.

B. FACS plots indicating gating strategy used for CLP analysis of BM (two femurs and two tibias) from KO and control mice. Data are representative of 3 independent experiments.

Figure S3.

A, B. Frequencies of donor ($CD45.2^+$) derived $CD19^+$, $CD11b^+$, $CD11b^+Ly6G^-$, $CD11b^+Ly6G^+$, $CD4^+$ and $CD8^+$ cells in the peripheral blood of lethally irradiated recipients that received total BM of either KO or control mice at 6 (**A**) and 18 (**B**) weeks of BMT. Data are pool of 2 independent experiments (n=10-14).

C. Frequencies of donor ($CD45.2^+$) derived $CD11b^+$, $Ter119^+$, $CD19^+$, $CD4^+$ and $CD8^+$ cells in the BM of lethally irradiated recipients that received total BM of either KO or control mice at 20 weeks of BMT. Data are pool of 2 independent experiments (n=8-14).

D. Absolute numbers of ST-HSC ($Flt3^-CD150^-CD48^-$ LSK) and MPP2 ($Flt3^-CD150^+CD48^+$LSK) subsets in the BM (two femurs and two tibias) of lethally irradiated recipients that received mixed BM of either KO (1:1) + competitor or control + competitor (1:1) mice. Data are pool of 2 independent experiments (n=5-6).

All data represent mean ± SEM. Two-tailed Student's *t* tests were used to assess statistical significance (*, $P < 0.05$; **, $P < 0.01$; ***, $P < 0.001$; ****, $P < 0.0001$; ns = not significant).

Figure S4.

A, B. Frequencies (**A**) and absolute numbers (**B**) of $CD11b^+$, $CD19^+$, $CD4^+$ and $CD8^+$ cells in the spleen of KO and control mice (n=15-20).

C. Frequencies of Lin^-, LK and LSK cells following *in-vitro* culture of purified Lin^- BM cells from KO and Control mice in the presence of HSPC cytokine cocktail for 24, 48 and 72 hours. Data are pool of two independent experiments (n=10-14).

D. Frequencies of LT-HSCs, ST-HSCs, MPP2, MPP3 and MPP4 subsets following *in-vitro* culture of purified Lin^- BM cells from KO and Control mice in the presence of HSPC cytokine cocktail for 24 and 48 hours. Data are pool of two independent experiments (n=10-14).

All data represent mean ± SEM. Two-tailed Student's *t* tests were used to assess statistical significance (*, $P < 0.05$).

Figure S5.

A. Frequencies of viable (propidium iodide$^-$ (PI^-)) cells at 36 and 64 hours of in-vitro culture in the presence of HSPC cytokine cocktail. Data are pool of 2 independent experiments.

B. Frequencies (**top**) and GMFI (**bottom**) of $BrdU^{+}$ Lin^{-} and LK cells following *in-vitro* culture of purified Lin^{-} BM cells from KO and Control mice in the presence of HSPC cytokine cocktail for 24 hours. Data are pool of two independent experiments (n=10).
C. Frequencies of $BrdU^{high}$ Lin^{-} and LK cells following *in-vitro* culture of purified Lin^{-} BM cells from KO and Control mice in the presence of HSPC cytokine cocktail for 24 hours. Data are pool of two independent experiments (n=10).
D, E. Frequencies of $Ki67^{+}$ Lin^{-} cells (**D**) and GMFI of $Ki67^{high}$ Lin^{-} and LK cells (**E**) in the BM of KO and Control mice (n=4).
F. Real time PCR data for *p16, p18, p21* and *p27* expression levels in Lin^{-} cells from the BM of KO and Control mice. Expression levels of target genes were normalized to HPRT levels. Data are representative of 3 independent experiments.
All data represent mean ± SEM. Two-tailed Student's *t* tests were used to assess statistical significance (*, $P < 0.05$; **, $P < 0.01$; ***, $P < 0.001$; ****, $P < 0.0001$).

Figure S6.
A-C. Real time PCR analysis of *c-Myc* and *Gfi1* mRNA expression in Lin^{-} cells (**A**), *Hoxa9* and *Cebp-α* mRNA expression in LK cells (**B**) and *Hoxa9* and *Cebp-α* mRNA expression in $LSKFlt3^{-}$ cells (**C**) in the BM cells from KO and control mice. Expression levels were normalized to HPRT levels. Data are representative of 3 independent experiments.
All data represent mean ± SEM. Two-tailed Student's *t* tests were used to assess statistical significance (*, $P < 0.05$; ****, $P < 0.0001$).

Figure S7.
A, B. Frequencies (**A**) and GMFI (**B**) of $H2DCF\text{-}DA^{+}$ (indicator of ROS levels) Lin^{-}, LK, LSK and MPP4 cells in the BM cells of KO (n=4) and Control (n=5) mice. All data represent mean ± SEM. Two-tailed Student's *t* tests were used to assess statistical significance.
